# EGFL6 regulates angiogenesis and osteogenesis in distraction osteogenesis via Wnt/β-catenin signaling

**DOI:** 10.1186/s13287-021-02487-3

**Published:** 2021-07-22

**Authors:** Junjie Shen, Yi Sun, Xuanzhe Liu, Yu Zhu, Bingbo Bao, Tao Gao, Yimin Chai, Jia Xu, Xianyou Zheng

**Affiliations:** Department of Orthopedic Surgery, Shanghai Jiao Tong University Affiliated Sixth People’s Hospital, Shanghai, 200233 PR China

**Keywords:** EGFL6, Osteogenesis, Bone marrow mesenchymal stem cells, Angiogenesis, Wnt/β-catenin, Distraction osteogenesis

## Abstract

**Background:**

Osteogenesis is tightly coupled with angiogenesis during bone repair and regeneration. However, the underlying mechanisms linking these processes remain largely undefined. The present study aimed to test the hypothesis that epidermal growth factor-like domain-containing protein 6 (EGFL6), an angiogenic factor, also functions in bone marrow mesenchymal stem cells (BMSCs), playing a key role in the interaction between osteogenesis and angiogenesis.

**Methods:**

We evaluated how EGFL6 affects angiogenic activity of human umbilical cord vein endothelial cells (HUVECs) via proliferation, transwell migration, wound healing, and tube-formation assays. Alkaline phosphatase (ALP) and Alizarin Red S (AR-S) were used to assay the osteogenic potential of BMSCs. qRT-PCR, western blotting, and immunocytochemistry were used to evaluate angio- and osteo-specific markers and pathway-related genes and proteins. In order to determine how EGFL6 affects angiogenesis and osteogenesis in vivo, EGFL6 was injected into fracture gaps in a rat tibia distraction osteogenesis (DO) model. Radiography, histology, and histomorphometry were used to quantitatively evaluate angiogenesis and osteogenesis.

**Results:**

EGFL6 stimulated both angiogenesis and osteogenic differentiation through Wnt/β-catenin signaling in vitro. Administration of EGFL6 in the rat DO model promoted CD31^hi^EMCN^hi^ type H-positive capillary formation associated with enhanced bone formation. Type H vessels were the referred subtype involved during DO stimulated by EGFL6.

**Conclusion:**

EGFL6 enhanced the osteogenic differentiation potential of BMSCs and accelerated bone regeneration by stimulating angiogenesis. Thus, increasing EGFL6 secretion appeared to underpin the therapeutic benefit by promoting angiogenesis-coupled bone formation. These results imply that boosting local concentrations of EGFL6 may represent a new strategy for the treatment of compromised fracture healing and bone defect restoration.

**Supplementary Information:**

The online version contains supplementary material available at 10.1186/s13287-021-02487-3.

## Background

Bone repair is a highly complex process of bone formation that recruits a diversity of cells and signaling pathways to achieve fracture healing and bone remodeling [1, 2]. As bone is highly vascularized connective tissue, it is not surprising that its vascular network serves both as a structural template and a key regulator of bone homeostasis [3, 4]. As with other tissues, blood vessels provide bone with nutrients and remove metabolites, but they also may be involved in the molecular signaling that occurs between angiogenesis and osteogenesis [4–8].

Recent studies found that functional epithelium of a specific capillary subtype called type H, which expresses high levels of CD31 and endomucin (CD31^hi^EMCN^hi^), mediates bone homeostasis in the bone microenvironment [9–11]. Accordingly, uncovering the potential molecular pathways that enhance type H vessel formation and osteogenesis can shed new light on the process of bone regeneration and repair [12].

To gain insight into natural fracture healing and to better understand large congenital bone defects, researchers have studied the molecular processes underlying distraction osteogenesis (DO). The temporal and spatial bone remodeling process of DO make it an ideal system to study the roles that angiogenesis and osteogenesis play in bone healing [8, 13]. DO is an innovative technique used by orthopedic surgeons to fix bone defects, and its validity has been verified in clinical and basic research [14–16].

DO comprises three phases: latency, distraction, and consolidation [17]. Following osteotomy and implantation of a distraction device, in the latency phase, the bone fragments are left undisturbed for 5–7 days during which a hematoma forms and bone regeneration begins. In the distraction phase, the device is engaged to gradually and continuously distract the bone segments until the desired length is achieved. In the consolidation phase, the distraction device is left in place to stabilize the bone, while the gap between bone fragments fills in with new bone and the resulting bony callus mineralizes until a sufficient level of bone regeneration is achieved [18, 19]. As successful bone regeneration largely depends on the blood supply [20, 21], it is not surprising, then, that DO is a highly vascular-dependent process that involves known and unknown angiogenic factors, including VEGF-A and epidermal growth factors (EGFs) among other known factors [22].

One factor that has recently captured researchers’ attention for its role in angiogenesis is epidermal growth factor-like domain-containing protein 6 (EGFL6) [23]. EGFL6 is a member of the EGF superfamily of proteins; it is also upregulated in tumorigenesis and epithelial-to-mesenchymal transition [24–27]. Xu et al. found that osteoblast-like cells secrete EGFL6 in a paracrine manner, triggering EC migration and angiogenesis through activation of the ERK pathway [28]. This suggests that direct crosstalk occurs between osteogenic cells and vascular ECs in the local bone environment. As osteoblasts are derived from bone mesenchymal stem cells (BMSCs), the crucial role of BMSCs in these processes has been proposed. However, little evidence exists for the angiogenesis-related mechanism of EGFL6’s action during the regulation of BMSC osteogenic differentiation.

In the present study, we tested the hypothesis that EGFL6 plays a central role in angiogenesis-associated osteogenesis. We observed that EGFL6 enhances angiogenesis through EC proliferation, migration, and vessel tube formation and that application of recombinant EGFL6 increases CD31 and EMCN—markers for type H blood vessels—expressed in human umbilical cord vein endothelial cells (HUVECs). We also provide evidence that EGFL6 could act directly in BMSC osteogenic differentiation to further support osteogenesis/angiogenesis. Finally, we show that EGFL6 functions partly via activation of the Wnt/β-catenin pathway. These results support our hypothesis that EGFL6 plays a key role in angiogenesis-associated osteogenesis during bone healing and that it could represent a new therapeutic target for facilitating bone repair and regeneration.

## Methods

### HUVEC cultures and functional assays

Recombinant EGFL6 protein was purchased from R&D Systems (Cat no.8638-EG-050, R&D Systems Inc., Minneapolis, MN, USA). HUVECs, a primary cell type used for in vitro studies of angiogenesis, were obtained from ScienCell Research Laboratories, Inc. (Catalog #8000; Carlsbad, CA, USA). HUVECs were cultured in endothelial cell medium (Catalog #1001; ScienCell Research Laboratories, Inc., Carlsbad, CA, USA) containing endothelial cell growth supplement (Cat #1052; ScienCell Research Laboratories, Inc., Carlsbad, CA, USA) and fetal bovine serum. HUVECs were maintained at 37 °C in a humidified incubator with an atmosphere of 5% CO_2_/95% air. EC proliferation assays were performed in 96-well culture plates using a cell proliferation assay kit (Cell Counting Kit-8 [CCK-8]; Dojindo Molecular Technologies, Inc., Rockville, MD, USA). CCK-8 is a colorimetric assay that measures the activity of cellular dehydrogenases, which are representative of overall cellular metabolic activity [29].

HUVECs (2 × 10^3^ cells/well) were seeded in medium supplemented with different concentrations of recombinant EGFL6 (0, 50, 200, 500 ng/ml). From day 0 to day 5, 10 μl of CCK8 solution were added to each well, and the samples were incubated for 2 h. Absorbance was read on a microplate reader at 450 nm, and optical density values were taken as a proxy indicator of cell proliferation.

EC migration assays were conducted in 24-well transwell culture plates having 8-μm pore filters (model no. 3428; Corning, Tewksbury, MA, USA). Briefly, HUVECs underwent serum starvation for 2 h, and then 8 × 10^4^ cells/well were seeded into the upper chamber of the transwell plate and incubated at 37 °C for 24 h. Cells remaining on the surface of the upper chamber were carefully scraped away with cotton swabs. Cells that had migrated to the lower chamber surface were fixed with 4% paraformaldehyde (PFA) for 30 min, stained with 0.1% crystal violet for 25 min, and then the stain was eluted briefly with 33% acetic acid. Absorbance at 570 nm was measured using a microplate reader.

For the scratch-wound assay, HUVECs were plated in 6-well culture plates and grown to confluence in EC medium (ScienCell Research Laboratories, Inc, Carlsbad, CA, USA) for 24 h. Then, the confluent monolayer was “scratched” with the same yellow plastic pipette tip (200 microliter). The scratch produced an initial cell-free gap over which cell migration could be monitored. After the scratch was made, the cultures were washed gently with PBS to remove non-adherent cells. The HUVEC cultures were then maintained in serum-free EC medium. The rate of cell-scratch-wound closure was determined by capturing images of the entire scratch at the indicated times using a CCD camera connected to an inverted phase-contrast microscope (Nikon Instruments Inc., Melville, NY, USA). Images were acquired at × 10 magnification and analyzed with ImageJ software (National Institutes of Health, Bethesda, MD, USA) [30].

The EC tube-formation assay (ECTFA) was conducted with HUVECS (3 × 10^3^ cells/well) seeded into 96-well culture plates precoated with Matrigel™ Matrix Growth Factor Reduced (Cat #356230; BD Biosciences, Franklin Lakes, NJ, USA). After incubation at 37 °C for 6 h, we used the Angiogenesis Analyzer plugin for ImageJ (National Institutes of Health, Bethesda, MD, USA) [30] and ImageJ to quantify the characteristics of the pseudo-capillary networks in the ECTFA [31]. We used an inverted phase-contrast microscope (Leica Microsystems GmbH, Wetzlar, Germany) to count the number of tube branches at × 4 magnification, measure the tube length (pixels), and count the numbers of capillary network meshes, nodes, and branches in five random fields per culture plate well.

### BMSC cultures and osteogenic differentiation assays

BMSCs were isolated from femurs of 4-week-old female Sprague-Dawley rats as previously described [32]. BMSCs were cultured in T25 tissue culture flasks. The culture medium was Gibco® α-MEM medium (Thermo Fisher Scientific, Waltham, MA, USA) containing 10% FBS and 1% penicillin/streptomycin. The BMSCs were incubated at 37 °C in a humidified incubator with a 5% CO_2_/95% air atmosphere. Cells from passage numbers 4-10 were used for all BMSC experiments.

The protocol used for BMSC osteogenic differentiation was described in detail in our previous study [33]. Briefly, when BMSC confluency reached 80%, α-MEM medium was replaced with osteogenic induction medium (OIM), which consisted of 1 nM dexamethasone, 50 μM L-ascorbic acid-2-phosphate, and 20 mM β-glycerophosphate. Different concentrations of EGFL6 (0, 50, 200, or 500 ng/ml) was added to OIM.

In order to determine whether WNT/β-catenin signaling was involved, OIM was supplemented with 200 ng/ml EGFL6 in the presence or absence of 0.3 μg/ml dickkopf-related protein 1 (DKK1; PeproTech, Cranbury, NJ, USA), an antagonist of Wnt/β-catenin signaling [34]. For staining extracellular mineral deposits, cells were fixed with 4% PFA, and then stained with Alizarin Red S (AR-S) for 10 min. To assay alkaline phosphatase (ALP) activity, osteoblasts were fixed with 4% PFA for 15 min, and then incubated with BCIP/NBT ALP Color Development Kit (C3206; Beyotime Biotechnology, Shanghai, China) according to the manufacturer’s protocol.

### Quantitative real-time PCR analysis

Total RNA was extracted using an EZ-press RNA Purification Kit (B0004D-100; EZBioscience, Roseville, MN, USA), and reverse transcription was performed with a cDNA Reverse Transcription Kit (EZBioscience, Roseville, MN, USA) according to the manufacturer’s protocol. We performed quantitative analysis using SYBR Green I Master Mix (EZBioscience, Roseville, MN, USA) and a LightCycler® 480 Real-time PCR system (Roche, Basel, Switzerland). The qPCR primers provided by BioTNT (Shanghai, China) are listed in Table 1.

**Table 1:** Real-time PCR primer sequences used in the study

Gene	Forward	Reverse
Human EMCN	5′ ACTAAGTGGATGTTGTTGGCT 3′	5′ AATAGTTCAGTTCAGCAAGGG 3′
Human CD31	5′ GGTGGAGTCTGGAGAGGACATT 3′	5′ GGGTGGCATTTGAGGTCATT 3′
Human Hif1a	5′ AGAAACCACCTATGACCTGCT 3′	5′ CGACTGAGGAAAGTCTTGCTA 3′
Human VEGF-A	5′ CAGAAGGAGGAGGGCAGAA 3′	5′ GTCTCGATTGGATGGCAGTAG 3′
Human GAPDH	5′ ATCCCATCACCATCTTCC 3′	5′ GAGTCCTTCCACGATACCA 3′
Rat EMCN	5′ AAGCACTGACAGAAACATCCA 3′	5′ ACTGTTGGTCGTTCCTTTAGG 3′
Rat CD31	5′ CACCGTGATACTGAACAGCAA 3′	5′ GTCACAATCCCACCTTCTGTC 3′
Rat EGFL6	5′ TAGTACCACTGTGCCTTCTCA 3′	5′ ACTCGTGTAGGAAGAGCAGAC 3′
Rat OCN	5′ CCTCACACTCCTCGCCCTATT 3′	5′ CCCTCCTGCTTGGACACAAA 3′
Rat OPN	5′ ATCTCCTAGCCCCACAGACC 3′	5′ TCCGTGGGAAAATCAGTGACC 3′
Rat ALP	5′ ACCATTCCCACGTCTTCACATTT 3′	5′ AGACATTCTCTCGTTCACCGCC 3′
Rat BMP2	5′ ACTCGAAATTCCCCGTGACC 3′	5′ CCACTTCCACCACGAATCCA 3′
Rat RUNX2	5′ ACTTCCTGTGCTCGGTGCT 3′	5′ GACGGTTATGGTCAAGGTGA 3′
Rat CXCR4	5′ CCTCTGAGGCGTTTGGTGCTC 3′	5′ TAGATGGTGGGCAGGAAGATC 3′
Rat CXCL2	5′ TCCTCAATGCTGTACTGGTC 3′	5′ TGAAGTCAACCCTTGGTAGG 3′
Rat Hif1a	5′ GGGTTATGAGCCAGAAGAACT 3′	5′ CCTGTGGTGACTTGTCCTTTA 3′
Rat VEGF-A	5′ TCAGGAGGACCTTGTGTGATC 3′	5′ CATTGCTCTGTACCTTGGGAA 3′
Rat COL1	5′ CATCGGTGGTACTAAC 3′	5′ CTGGATCATATTGCACA 3′
Rat GAPDH	5′ GGCATGGACTGTGGTCATGAG 3′	5′ TGCACCACCAACTGTTAGC 3′

All primers provided by BioTNT (Shanghai, China)

### Western blot analysis

Cells were diluted at a 1:4 ratio with loading buffer (5X), and then heated at 95 °C for 5 min. Protein extracts were separated by 7.5%, 10%, or 15% SDS-PAGE and blotted onto PVDF membranes (Millipore, Billerica, MA, USA). Subsequently, the membranes were blocked with 6% nonfat milk for 2 h. The PVDF membranes were then incubated overnight with primary antibodies against Hif1a (1:2000, no. 14179, Cell Signaling Technology [CST]), VEGF-A (1:1000, ab1316, Abcam), EMCN (1:1000, ab96315, Abcam), CD31 (1:2000, ab32457, Abcam), BMP2 (1:1000, ab214821, Abcam), Runx2 (1:2000, no. 8486, CST), CXCR4 (1:2000, ab124824, Abcam), p-β-catenin (1:2000, no. 9561, CST), β-catenin (1:2000, no. 8480, CST), non-phospho (active) β-catenin (Ser33/37/Thr41) (D13A1) (1:2000, no. 8814, CST), p-AKT (1:2000, no. 9271, CST), AKT (1:2000, no. 9272, CST), Erk1/2 (1:2000, AF0155, Affinity), phospho-GSK-3β (Ser9) (D85E12) XP® (pGSKβ 1:1000, no. 5558, CST), and GAPDH (1:2500, no. 5174, CST) (Table 2). The membranes were then washed three times in TBST buffer, and then incubated with species-appropriate HRP-conjugated secondary antibodies for 1 h at RT. The immunoreactive bands were visualized using ECL kit (no. SQ201, EpiZyme Biotechnology Ltd., Shanghai, China) and detected using a ChemiDoc Imaging System (BioRad, Hercules, CA, USA). GAPDH was used as the protein loading control. All immunoblots presented in the figures were cropped from the originals.

**Table 2:** Antibodies used in the study

Antibody	Purpose	Dilution	Product ID and manufacturer
Anti-Hif1a	Western blot	1:2000	#14179, Cell Signaling Technology [CST], Danvers, MA, USA
Anti-VEGF-A	Western blot	1:1000	ab1316, Abcam, Cambridge, UK
Anti-EMCN	Western blot	1:1000	ab96315, Abcam, Cambridge, UK
Anti-CD31	Western blot	1:2000	ab32457, Abcam, Cambridge, UK
Anti-BMP2	Western blot	1:1000	ab214821, Abcam, Cambridge, UK
Anti-RUNX2	Western blot	1:2000	#8486, CST, Danvers, MA, USA
Anti-CXCR4	Western blot	1:2000	ab124824, Abcam, Cambridge, UK
Anti-p-β-catenin	Western blot	1:2000	#9561, CST, Danvers, MA, USA
Anti-β-catenin	Western blot	1:2000	#8480, CST, Danvers, MA, USA
Anti-active β-catenin	Western blot	1:2000	#8814, CST, Danvers, MA, USA
Anti-p-GSK3β	Western blot	1:2000	#5558, CST, Danvers, MA, USA
Anti-p-AKT	Western blot	1:2000	#9271, CST, Danvers, MA, USA
Anti-AKT	Western blot	1:2000	#9272, CST, Danvers, MA, USA
Anti-Erk1/2	Western blot	1:2000	AF0155, Affinity Biosciences Ltd., Cincinnati, OH, USA
Anti-GAPDH	Western blot	1:2500	#5174, CST, Danvers, MA, USA
Anti-OCN	IHC	1:200	ab198228, Abcam, Cambridge, UK
Anti-VEGF-A	IHC	1:200	Ab1316, Abcam, Cambridge, UK
Anti-active β-catenin	IHC	1:200	#8814, CST, Danvers, MA, USA
Anti-RUNX2	IHC	1:250	#8486, CST, Danvers, MA, USA
Anti-EMCN	IHC	1:100	sc-65495, Santa Cruz Biotechnology Inc., Dallas, TX, USA
Anti-CD31	IHC	1:100	ab24590, Abcam, Cambridge, UK
Alexa Fluor®	IHC	1:500	Jackson Research Inc., West Grove, PA, USA

*IHC* immunohistochemistry

### Rat distraction osteogenesis model

Procedures for the animal distraction model were approved by the Animal Care and Use Committee of Shanghai Jiao Tong University Affiliated Sixth People’s Hospital. Twenty-eight male Sprague-Dawley rats were equally divided in control and EGFL6 groups. Tibia DO surgery was performed according to previously established protocols [19]. Briefly, the rat was anesthetized with 4% chloral hydrate (0.7 ml/100 g), surgical area was shaved, cleaned with 75% alcohol, and a 30-mm incision was made over the middle part of the tibia. At the midshaft of the tibia, a 5-mm wide defect was made, producing two bone segments. A monolateral external fixator (Xinzhou Company, Tianjin, China) was mounted onto the proximal and distal bone segments with four stainless steel pins. The incisions were then sutured closed layer by layer. The timeline of events is shown in Fig. 5a.

Distraction was performed in three phases: (1) a 5-day latency phase, (2) a 10-day distraction or active lengthening phase, and (3) a 4-week consolidation phase. In the latency phase, the defect in the tibia was left undisturbed in order to initiate the early stages of bone healing. In the distraction phase, the distraction gap was infused with 0.5 ml of recombinant EGFL6 protein (200 ng/ml) or an equivalent volume of sterile PBS (control) every 2 days. Tibia specimens were harvested in the second and fourth week of the consolidation phase (*n* = 7 per group).

### Immunohistochemistry, histology, and histomorphometry

For immunofluorescence staining, cell samples were fixed using 4% PFA for 15 min, permeabilized with 0.3% Triton X-100 for 15 min, and blocked with 5% BSA in phosphate-buffered saline–Tween20 (PBST, 0.1% Tween20) for 1 h at RT. Tissue sections were processed similarly. After washing with PBS, samples were incubated overnight at 4 °C with primary antibodies: anti-β-catenin (1:200, ab32572, Abcam), anti-RUNX2 (1:250, no. 8486, CST), anti-EMCN (1:100, sc-65495, Santa Cruz Biotechnology), and anti-CD31 (1:100, ab24590, Abcam). The next day, samples were washed gently with PBS for three times and incubated with species-appropriate Alexa Fluor® secondary antibodies (1:500, Jackson Research) for 1 h at RT (Table 2). Nuclei were counterstained with DAPI (Sigma-Aldrich, St. Louis, MO, USA), and actin filaments were labeled with TRITC phalloidin (Yeasen Biotechnology, Shanghai, China). Immunofluorescence in sections was imaged with a Leica epifluorescence microscope (Leica, Wetzlar, Germany), and images were acquired for analysis. The intensity of immunofluorescent staining was quantified using ImageJ software (National Institutes of Health, Bethesda, MD, USA) [30].

For histological analysis, tibia specimens were decalcified in 10% EDTA for 4 weeks, progressively dehydrated with ethanol to 100% ethanol, cleared in xylene, embedded in paraffin, and sectioned at a thickness of 5 μm. The decalcified tibia sections were stained with hematoxylin and eosin (HE), Masson’s trichrome stain, or Safranin O/Fast green stain. Immunohistochemical (IHC) staining was performed using primary antibodies against osteocalcin (OCN) (1:200, ab198228, Abcam), followed by species-appropriate HRP-conjugated secondary antibody. Average optical density was measured in five random visual fields per section using ImageJ software (National Institutes of Health, Bethesda, MD, USA).

### Digital radiography and micro-computed tomography

Starting with the first week of the consolidation phase, animals underwent weekly X-ray imaging of the distraction gap. The rats were anesthetized with general anesthesia during the X-rays. At the end of the consolidation phase, the rats were killed with an overdose of 4% chloral hydrate, and the tibias were harvested for three-dimensional (3D) reconstructions using micro-CT analysis. We used a micro-CT in-Vivo SkyScan™ (SkyScan-1176; Bruker Corporation, Billerica, MA, USA) and a voxel size of 18 μm for all three spatial dimensions. Bone volume/total volume (BV/TV) and bone mineral density (BMD) were analyzed using CTan software (v1.13.2.1, Skyscan, Bruker Corporation, Billerica, MA, USA) and CTvol software (v2.4.0, Skyscan, Bruker Corporation, Billerica, MA, USA).

### Statistical methods

All quantitative data are presented as means ± standard deviation (SD). SPSS 22.0 (IBM Corp. Released 2013. IBM SPSS Statistics for Windows, Version 22.0. Armonk, NY: IBM Corp.) was used for statistical analyses. Two-tailed Student’s *t* tests were used for comparisons of two groups, and one-way ANOVA was used for multiple groups followed by Dunnett’s multiple comparisons test. All experiments were repeated at least three times to support reproducibility. *P* < 0.05 was accepted as statistically significant.

## Results

### EGFL6 acts on endothelial cells to promote CD31^hi^EMCN^hi^ type H endothelium formation in vitro

**Fig. 1:**
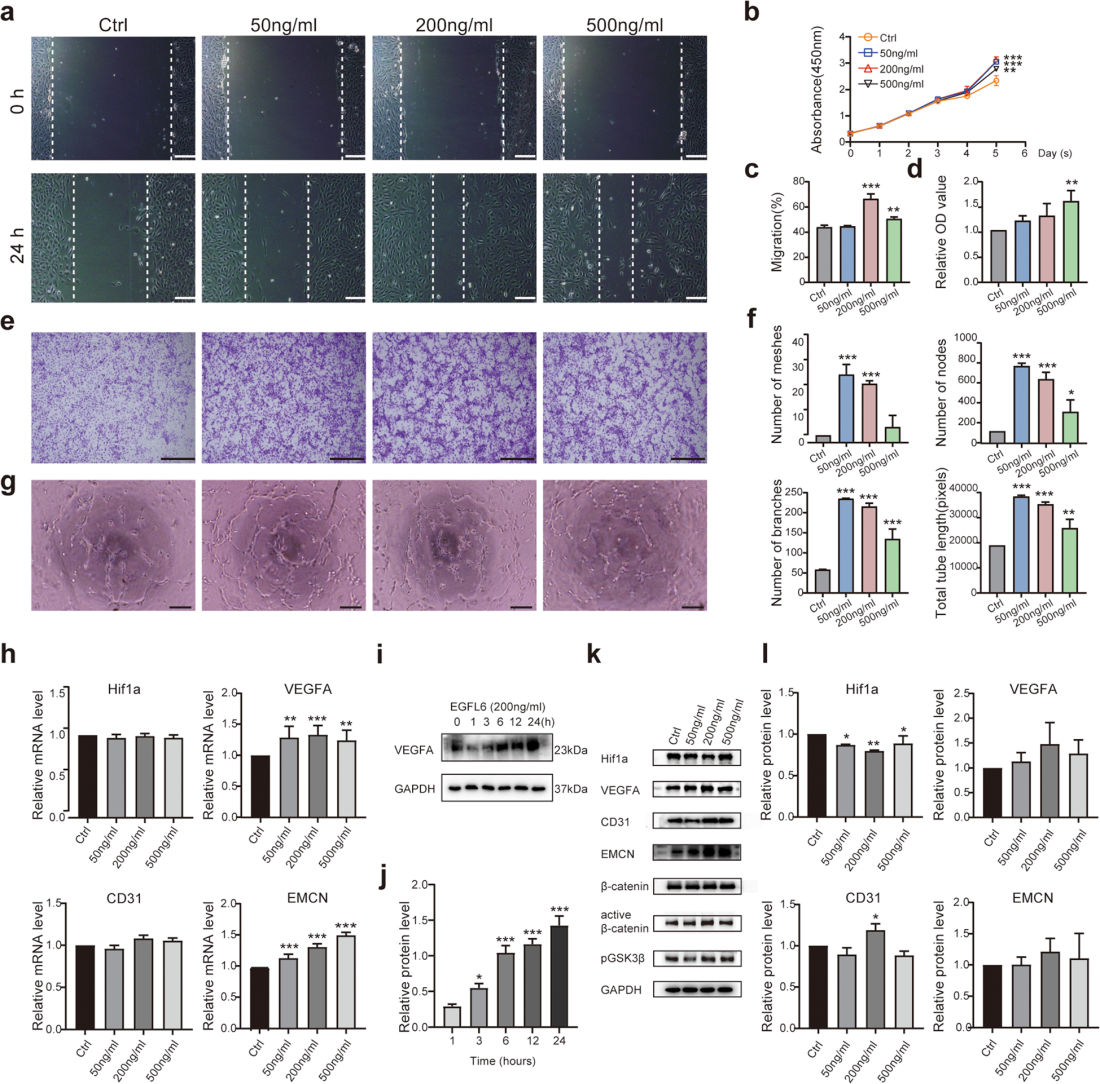
Effects of different concentrations of EGFL6 protein on human umbilical cord vein endothelial cell (HUVEC) angiogenesis in vitro. **a** Phase-contrast images of HUVEC cultures treated with EGFL6 showing cell migration in the scratch-wound assay at the indicated times. Vertical dashed lines (white) demarcate the border between the wavefront of migrating cells and scratched area that was initially void of cells. **b** Quantitation (mean ± SD) of cell proliferation in response to EGFL6 (CCK-8 assay). **c** Mean percentage of cells migrating as a function of EGFL6 concentration in the scratch-wound assay. **d, e** Crystal violet-stained HUVECs that migrated in the transwell assay. Optical density (OD) of staining is relative to untreated control cells (**e**). **f** Relative quantification of capillary-like structures formed by HUVECs cultured with EGFL6 in the tube-formation assay. Values are relative to control values. **g** Phase-contrast images of HUVECs cultured with EGFL6 in the tube-formation assay. **h** Expression levels of Hif1a, VEGF-A, CD31, and EMCN genes in HUVECs treated with EGFL6 for 1 day, as evaluated by RT-PCR. The housekeeping gene GAPDH served as an internal control. **i, j** Quantitation of VEGF-A protein concentration in HUVECs treated with EGFL6 (200 ng/ml) for the indicated times. **k, l** Western blots of lysates from HUVECs treated with EGFL6. Blots were probed with antibodies against angiogenesis markers (Hif1a, VEGF-A, CD31, EMCN) and pathway markers (β-catenin, pβ-catenin, active β-catenin, and pGSK3β). GADPH is the loading control. Significant differences among groups were determined by one-way ANOVA and post hoc Dunnett’s test; *p < 0.05; **p < 0.01; and ***p < 0.001. All immunoblots were cropped from the original here and in subsequent figures. Experimental HUVECs were treated with the indicated EGFL6 concentrations. Control and experimental conditions for all functional assays were the same, except controls lacked EGFL6. Histogram values are based on three replicated experiments, and error bars are SD here and in all subsequent figures. Scale bars for **a, e, g**, 250 μm

Angiogenesis involves proliferation and migration of ECs and ultimately capillary tube formation and upregulation of some angiogenesis-related factors in vivo [35]. We used HUVECs to determine how EGFL6 affects HUVEC proliferation; we measured cell proliferation in vitro with the CCK-8 assay. EGFL6 promoted cell proliferation in a mostly concentration-dependent manner. After 5 days of EGFL6 treatment, 50 and 200 ng/ml EGFL6 were the most effective concentrations to promote cell proliferation (Fig. 1b).

Next, we determined whether EGFL6 influences cell migration. Cell migration as assessed in the scratch-wound assay (Fig. 1a, c) and transwell assay (Fig. 1d, e) indicated that the migration of EGFL6-treated HUVECs was significantly enhanced compared with that of untreated control cells. In addition, EGFL6-treated HUVECs displayed an enhanced ability to induce capillary tube formation (Fig. 1f, g), supporting the finding that EGFL6 is a proangiogenic factor [28]. We also noticed differences in the optimal EGFL6 concentrations between assays, with 200 and 500 ng/ml EGFL6 being more effective in the migration assays (scratch assay and transwell assay) and the 50 ng/ml concentration, and to a lesser extent 200 ng/ml EGFL6, being more effective for capillary tube formation.

To investigate the mechanism of EGFL6’s angiogenic effects on HUVECs, we measured the expression of VEGF-A, one of the master regulators of vascular growth [1, 36, 37]. In HUVECs maintained in culture, RT-PCR and western blot analyses of cell lysates of EGFL6-treated cells demonstrated that VEGF-A mRNA and protein levels were both elevated by EGFL6 (Fig. 1h–j). VEGF-A expression levels increased significantly with increasing culturing time (Fig. 1i, j). In addition, RT-PCR analysis for CD31 and EMCN, two markers for type H vessels [12], revealed that CD31 and EMCN mRNA expression levels were also upregulated 24 h after treatment with EGFL6 (Fig. 1h).

Western blot analysis revealed a similar trend, with EGFL6-treated HUVECs expressing higher levels of CD31 and EMCN proteins, particularly when treated with a higher concentration of EGFL6 (200 ng/ml) (Fig. 1k, l). These results indicate that EGFL6 has specialized functional properties in promoting angiogenesis and inducing the expression of CD31 and EMCN, which characterizes type H vessels [12].

### EGFL6 enhances osteogenic differentiation of BMSCs

**Fig. 2:**
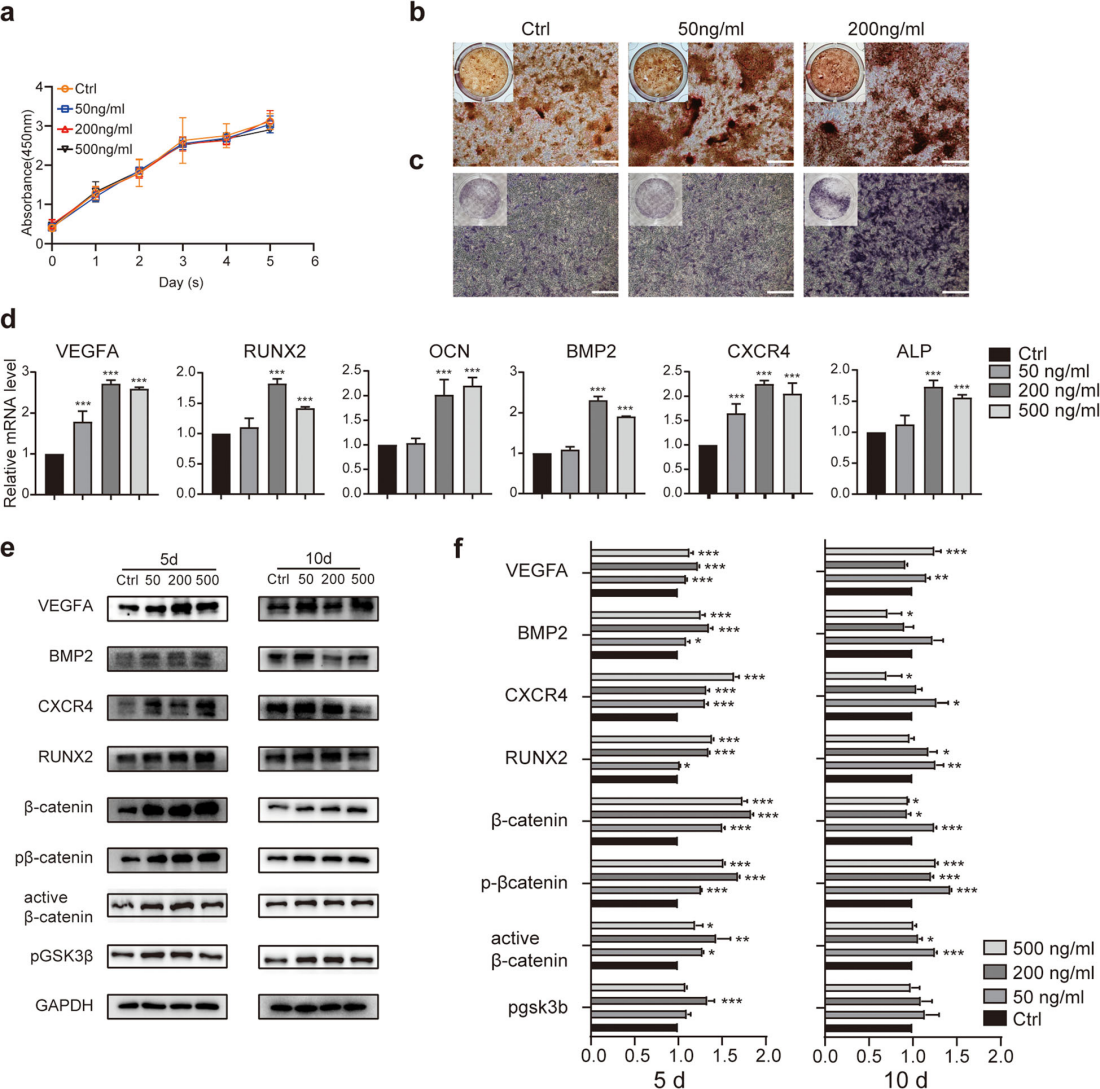
EGFL6 treatment enhances osteogenic differentiation of rat bone marrow mesenchymal stem cells (BMSCs) in vitro. **a** BMSC viability after treatment with different concentrations of EGFL6, as assessed by the CCK-8 assay. Values are means±SD. **b** Images of Alizarin Red S (AR-S)-stained BMSCs treated with EGFL6, showing increased mineralization (rust-colored deposits). Osteogenic differentiation of BMSCs was examined on day 14. **c** Images of alkaline phosphatase (ALP)-stained BMSCs treated with EGFL6. Osteogenic differentiation of BMSCs was examined on day 3. Insets in **b** and **c** show low-magnification images of entire culture well. Scale bars, 250 μm. BMSCs were treated with different concentrations of EGFL6 for 5 or 10 days. **d** Expression levels of angiogenesis- and osteogenesis-related markers in BMSCs following treatment with/without EGFL6 for 5 days, as evaluated by RT-PCR. The housekeeping gene GAPDH served as an internal control. **e** Western blots of lysates from cultured BMSCs treated with/without EGFL6 for 5 or 10 days. Blots were probed with antibodies against different markers for angiogenesis (VEGF-A), osteogenesis (BMP2, CXCR4, RUNX2), and the Wnt/b-catenin signaling pathway (b-catenin, pb-catenin, active β-catenin, and pGSK3β). **f** Quantitation of expression of angiogenesis-, osteogenesis-, and pathway-related marker proteins in panel **e**. Significant differences among groups were determined by one-way ANOVA and post hoc Dunnett’s test; *p < 0.05; **p < 0.01; and ***p < 0.001

EGFL6 has been shown to be highly expressed in osteoblastic-like cells [28, 38]. This, together with our findings that EGFL6 enhances angiogenesis, prompted us to investigate whether EGFL6 could directly increase the osteogenic capacity of BMSCs. To address this possibility, we treated BMSCs maintained in culture with OIM supplemented with different concentrations of EGFL6. As measured in the CCK-8 cell proliferation assay, EGFL6 treatment (50, 200, or 500 ng/ml EGFL6 for 1-5 days) failed to affect cell proliferation compared to the untreated control (*p* > 0.05) (Fig. 2a). Thus, EGFL6 at the tested concentrations did not affect BMSC proliferation, and it had no significant cytotoxicity.

Next, we assessed the ability of EGFL6 to increase mineralization deposits by measuring ALP activity and calcium deposition in BMSCs by staining the cells for ALP and with AR-S. Compared with untreated control cells, EGFL6-treated BMSCs had more abundant mineralized nodules and significantly denser AR-S staining, indicating increased accumulation of calcium (Fig. 2b, c). Calcium deposition was EGFL6 concentration dependent, with more deposition at higher EGFL6 concentrations.

**Fig. 3:**
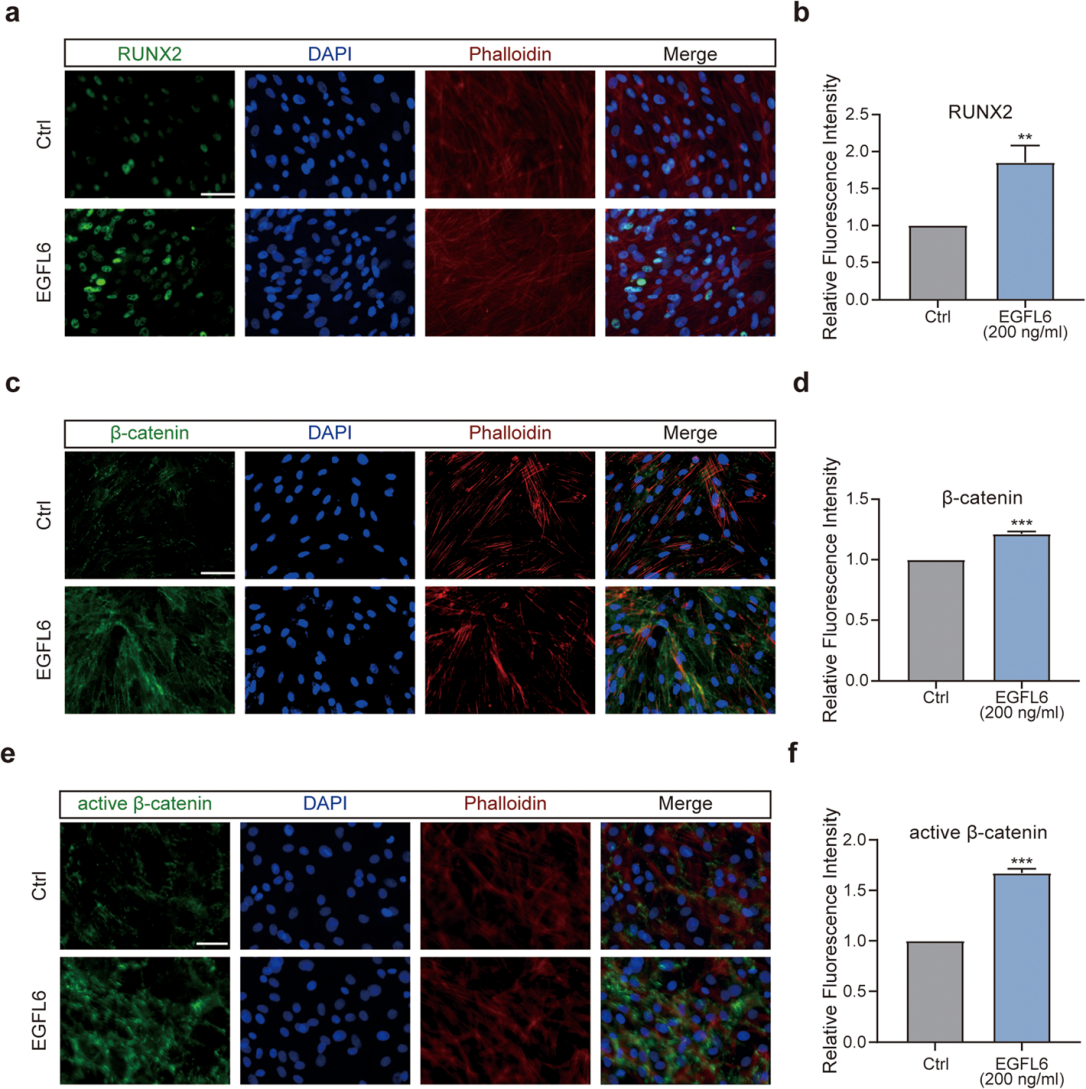
EGFL6 treatment enhances osteogenic differentiation of rat bone marrow mesenchymal stem cells (BMSCs) in vitro*.* **a, c, e** Immunofluorescent images of EGFL6-treated BMSCs stained for the osteogenic-specific protein RUNX2 (**a**), and pathway-specific protein β-catenin (**c**) and active β-catenin (**e**). Cells were counterstained with the nuclear stain DAPI (blue) and the cytoskeleton stain phalloidin (red). Scale bars, 100 μm. **b, d, f** Quantitation of mean relative levels of RUNX2 (**b**), β-catenin (**d**), and active β-catenin (**f**) in BMSCs treated with EGFL6 (200 ng/ml). Significant differences between experimental and control groups were evaluated by Student *t* tests; *p < 0.05; **p < 0.01; and ***p < 0.001

To further investigate how EGFL6 affects osteogenic differentiation of BMSCs, we measured the expression of osteogenic-related genes and their proteins in cultured BMSCs 5 and 10 days after EGFL6 treatment by RT-PCR and western blotting (Fig. 2d–f). After 5 days of EGFL6 treatment, the expression levels of VEGF-A and osteogenic markers (RUNX2, CXCR4, and BMP2) were all significantly increased. EGFL6 concentrations of 200 ng/ml and above had a stronger effect. This increasing trend was also observed after 10 days of EGFL6 treatment. Notably, there was a less obvious dose-dependent relationship at 10 days. The possible reason was that the osteogenesis process develops faster with higher concentrations of EGFL6. Immunofluorescence staining for RUNX2 confirmed this trend, as higher expression levels of RUNX2 was detected after EGFL6 treatment (Fig. 3a, b).

We also measured the expression of Wnt pathway-related markers in BMSCs following EGFL6 treatment for 5 or 10 days (Fig. 2e, f). It is well known that an increase in phosphorylated β-catenin levels means that it is being targeted for ubiquitin-associated degradation; this then inhibits the canonical Wnt pathway. Thus, we quantified active β-catenin and pGSK3β levels in EGFL6-treated BMSCs by western blotting and found higher expression after EGFL6 treatment (Fig. 2e, f). These results provide additional evidence that EGFL6 acts through the Wnt pathway in BMSCs. Collectively, these results provide supporting evidence for the hypothesis that EGFL6 stimulated osteogenic differentiation along with angiogenesis.

### EGFL6-mediated Wnt/β-catenin signaling may regulate angiogenesis and osteogenesis

One potential mechanism through which EGFL6 promotes angiogenesis and osteogenesis is Wnt/β-catenin signaling [39, 40]. The Wnt/β-catenin pathway has been shown to control the cell fate of mesenchymal stem cells, causing them to become osteoblasts [39, 40]. Also, Wnt/β-catenin signaling appears to play an important role in tumor angiogenesis [41–43]. Thus, we investigated the expression of key factors involved in several signaling pathways after EGFL6 treatment.

Western blot analysis revealed that EGFL6 treatment enhanced the expression of β-catenin and active β-catenin in cultured HUVECs and BMSCs (Figs. 1k and 2e, f). Furthermore, in vitro experiments of BMSC osteogenic differentiation showed that β-catenin and active β-catenin immunofluorescence staining was significantly enhanced 5 days after osteogenic induction with EGFL6 at concentrations greater than 200 ng/ml (Fig. 3c–f). This result suggests that the Wnt/β-catenin pathway may be involved in EGFL6-mediated angiogenesis and osteogenesis. In addition, western blot analysis suggested that EGFL6 treatment altered the expression levels of Akt, p-Akt, and P-ERK1/2 to varying degrees in HUVECs and BMSCs (Additional file 1a-c), indicating that other signaling pathways may also be involved in EGFL6-mediated angiogenesis and osteogenesis.

### DKK1 partially inhibits BMSC osteogenesis activated by EGFL6

**Fig. 4:**
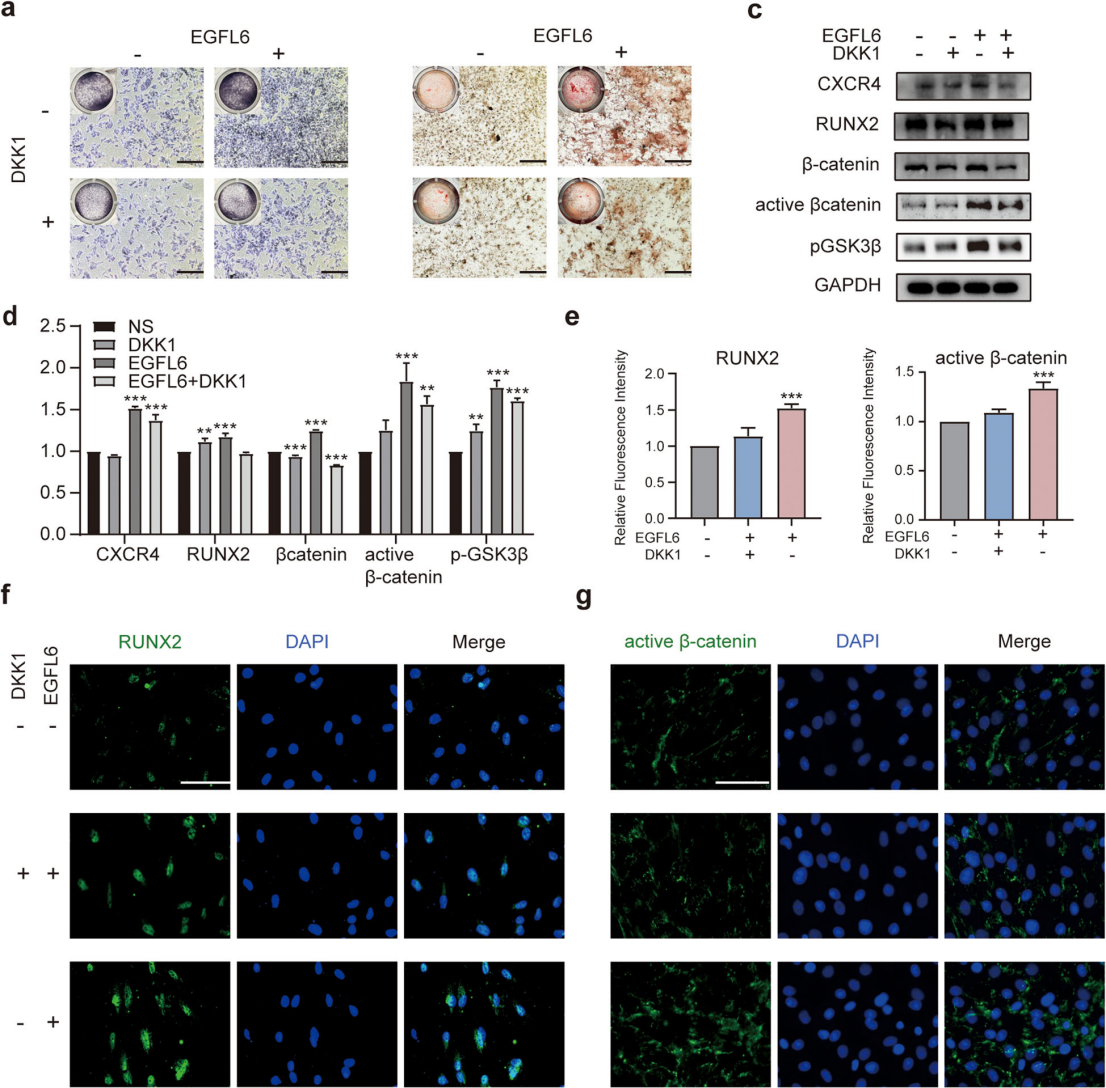
Dickkopf-related protein 1 (DKK1) partially suppresses EGFL6-enhanced BMSC osteogenesis in vitro. **a** Light micrographs of ALP-stained BMSC cultures on day 3 of differentiation. BMSCs were treated with 200 ng/ml EGFL6 to enhance osteogenic differentiation, and then supplemented with/without 0.3 μg/ml DKK1. Insets show low-magnification images of entire culture well. Scale bars, 250 μm. **b** AR-S staining of differentiated BMSCs showing mineralization (red) on day 14 after DKK1 application. Insets show low-magnification images of entire culture well. Scale bars, 250 μm. **c** Western blots showing the expression of osteogenic-specific and Wnt/β-catenin signaling-related proteins in BMSCs treated with/without EGFL6 and with/without DKK1. GADPH is the loading control. **d** Quantitation of osteogenic-specific and Wnt/β-catenin signaling-related proteins normalized to control condition (NS; black-colored bars). **e, f, g** Immunofluorescent images of BMSCs stained for RUNX2 (green) or active β-catenin (green). BMSCs were cultured with 200 ng/ml EGFL6 to enhance BMSC osteogenesis, and then treated with/without 0.3 μg/ml DKK1, an antagonist of Wnt/β-catenin signaling. Scale bar, 100 μm. Quantitation of RUNX2 or active β-catenin immunofluorescent staining showing mean relative fluorescence of DKK1 + EGFL6 (blue-colored bars) and EGFL6 alone (pink-colored bars) conditions normalized to control fluorescence (no DKK1, no EGFL6; gray-colored bars). Significant differences were evaluated by one-way ANOVA and post hoc Dunnett’s tests for all panels; *p < 0.05; **p < 0.01; and ***p < 0.001

To further investigate the involvement of the Wnt/β-catenin signaling pathway in EGFL6-enhanced BMSC osteogenesis, we evaluated how inhibition of this pathway with DKK1, a Wnt signaling pathway inhibitor, affects osteogenesis in BMSCs treated with EGFL6 in OIM medium. ALP and AR-S staining of BMSC cultures revealed higher ALP expression and calcium deposition, respectively, in EGFL6-treated cultures than in those treated with EGFL6 and DKK1 (Fig. 4a, b). Also, DKK1 application significantly decreased β-catenin and active β-catenin expression levels in BMSCs enhanced with EGFL6, compared to BMSCs cultured without EGFL6 and DKK1 (control) and BMSCs cultured with EGFL6 alone. Blocking the Wnt/β-catenin pathway with DKK1 partially reversed the EGFL6-mediated upregulation of osteogenic proteins (CXCR4, RUNX2), as demonstrated by western blotting (Fig. 4c, d) and immunofluorescence staining (Fig. 4e–g). These results indicate that the EGFL6-enhanced differentiation of BMSCs into osteoblast-like cells is mediated by Wnt/β-catenin signaling.

### EGFL6 accelerates osteogenesis and promotes the formation of CD31^hi^EMCN^hi^-positive type H vessels in a rat DO model

**Fig. 5:**
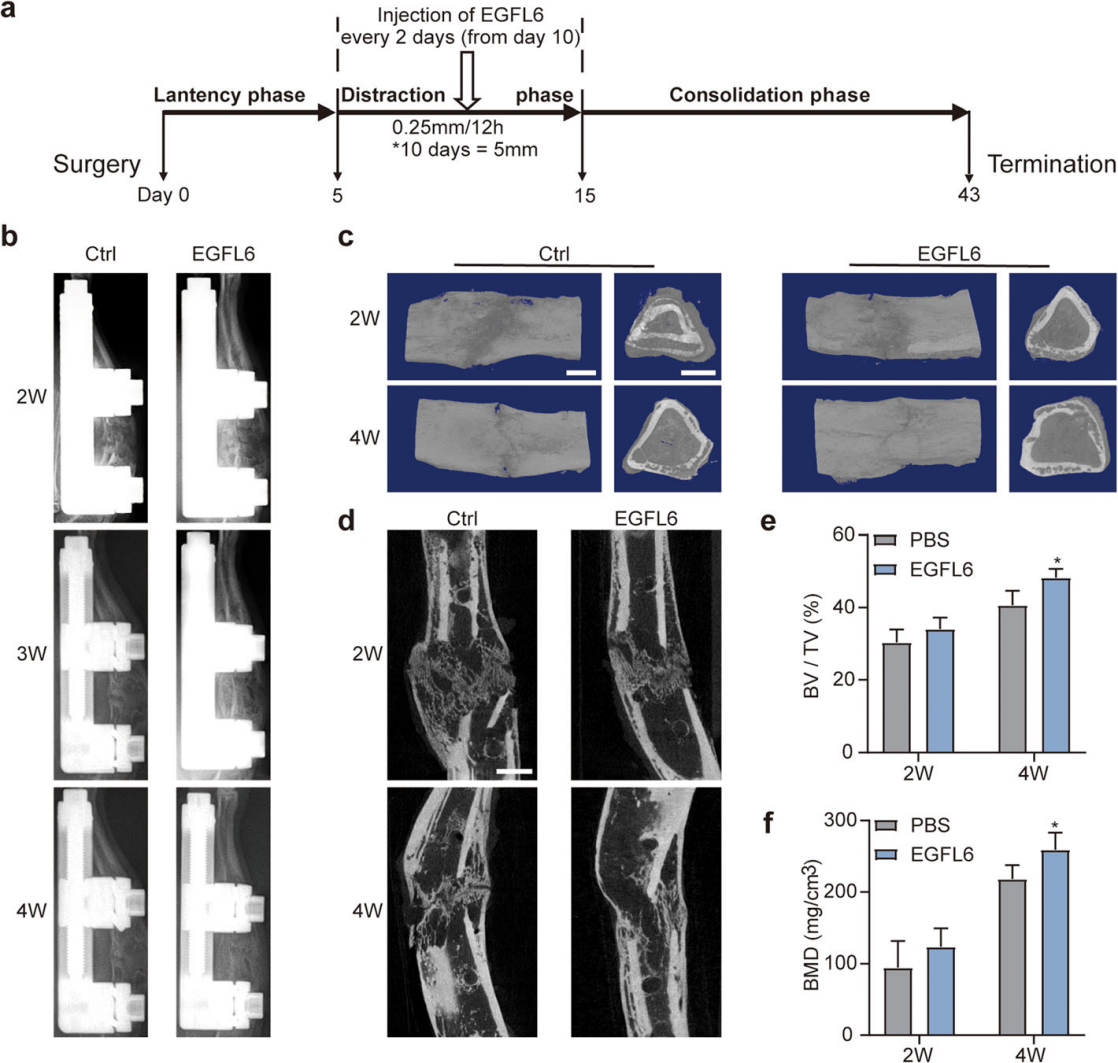
Locally applied EGFL6 accelerates bone formation and consolidation in a rat model of tibia distraction osteogenesis (DO). **a** Overall schematic diagram illustrating the study design. DO was performed in three phases as indicated. Midway through the distraction phase on day 10, recombinant EGFL6 (200 ng/ml), or an equivalent volume of sterile PBS (control), was infused into the distracted area and then infused again every 2 days until the end of the distraction phase on day 15. Distraction was performed at a rate of 0.25 mm per 12 h. Asterisk (*) in **a** indicates that the tibia bone fragments were distracted for a total of 5 mm over a period of 10 days. **b** X-ray images (lateral view) of the distracted bones from representative cases after 2, 3, and 4 weeks of consolidation. Bright white angular areas in images are the densities of the metal monolateral external fixator. **c, d** Three-dimensional reconstructions (**c**) and internal longitudinal profiles (**d**) derived from micro-CT of distracted tibia bones from representative cases of EGFL6-treated and control rats after 2 and 4 weeks of consolidation. Light areas show the increased bone-tissue mineralization. **e, f** Quantitation analysis of bone-tissue mineralization showing the mean (±SD) percentage bone volume/total tissue volume (BV/TV) and mean (±SD) bone mineral density (BMD) in EGFL6-treated and control rats. Mineralization parameters were calculated from the micro-CT image data. Significant differences were evaluated by one-way ANOVA with post hoc Dunnett’s tests. *p < 0.05

We used a rat DO model to evaluate the effect of EGFL6 on bone regeneration and angiogenesis in vivo. The surgical procedure and treatment schedule for the rat DO model is schematically diagrammed in Fig. 5a and shown in Additional file 2.

Over the course of 4 weeks, increased callus was predominantly associated with EGFL6 infusion (200 ng/ml every 2 days) into the distraction gap during the distraction phase, as seen in radiographs of the rat tibia of control (PBS infusion) and experimental groups (Fig. 5b). The callus in the EGFL6-treated group was larger and denser than that in the PBS-treated control group. Figure 5c and d show 3D reconstructions and internal longitudinal profiles of the regenerated bone in the distracted area 2 and 4 weeks after consolidation. These morphological data were obtained through micro-CT analysis. Both BMD and BV/TV values were higher in the EGFL6-treated group than in the PBS group (Fig. 5e, f), indicating that EGFL6 enhances bone regeneration.

**Fig. 6:**
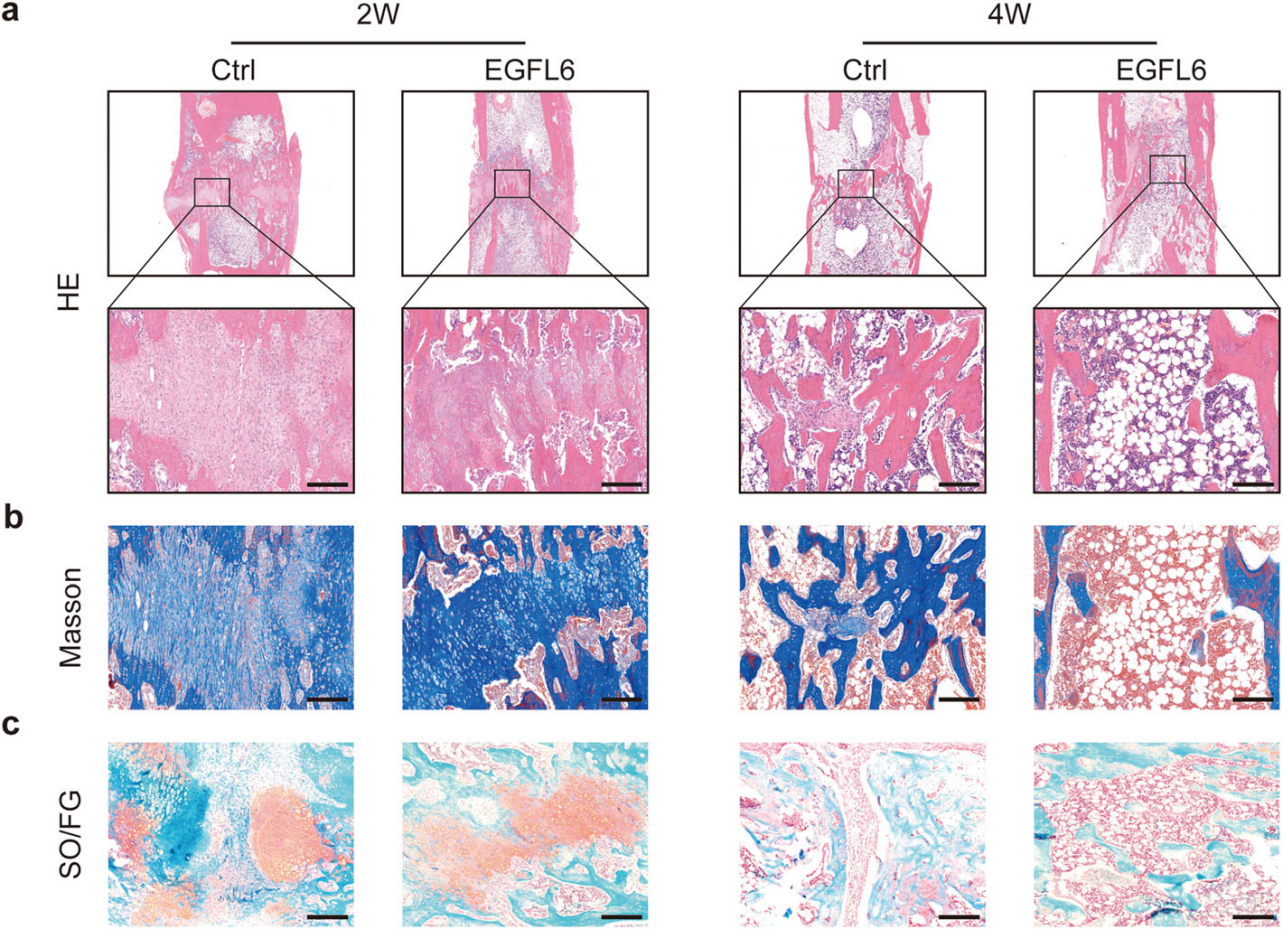
EGFL6 stimulated formation of bone after consolidation for 2 and 4 weeks in a rat tibia DO model. **a–c** Images of histological sections of regenerated bone obtained from the distraction zone (boxed areas in **a**) of rats treated with EGFL6 or PBS (control). Sections were stained with hematoxylin and eosin (HE) (**a**), Masson trichrome stain (**b**), or Safranin O/Fast green stain (**c**) in order to indicate newly formed trabecular bone, fibrous tissue, and cartilaginous tissue, respectively, in the distracted area. Scale bars, 200 μm

Histological and immunohistological sections of the regenerated bone in distracted tibias were analyzed after 2 and 4 weeks of consolidation in EGFL6-treated and control rats. During the distraction phase, a central fibrous interzone rich in fibroblasts and chondrocyte-like cells formed as the callus was stretched. In the consolidation phase, we observed a high density of proliferating osteoblasts bridging the fibrous interzone from either side of the gap, forming along capillaries and vascular sinuses (Fig. 6). These osteoblasts underwent primary mineralization, leading to the formation of columns of bone resembling the morphology of stalagmites and stalactites, as described previously [44, 45].

**Fig. 7:**
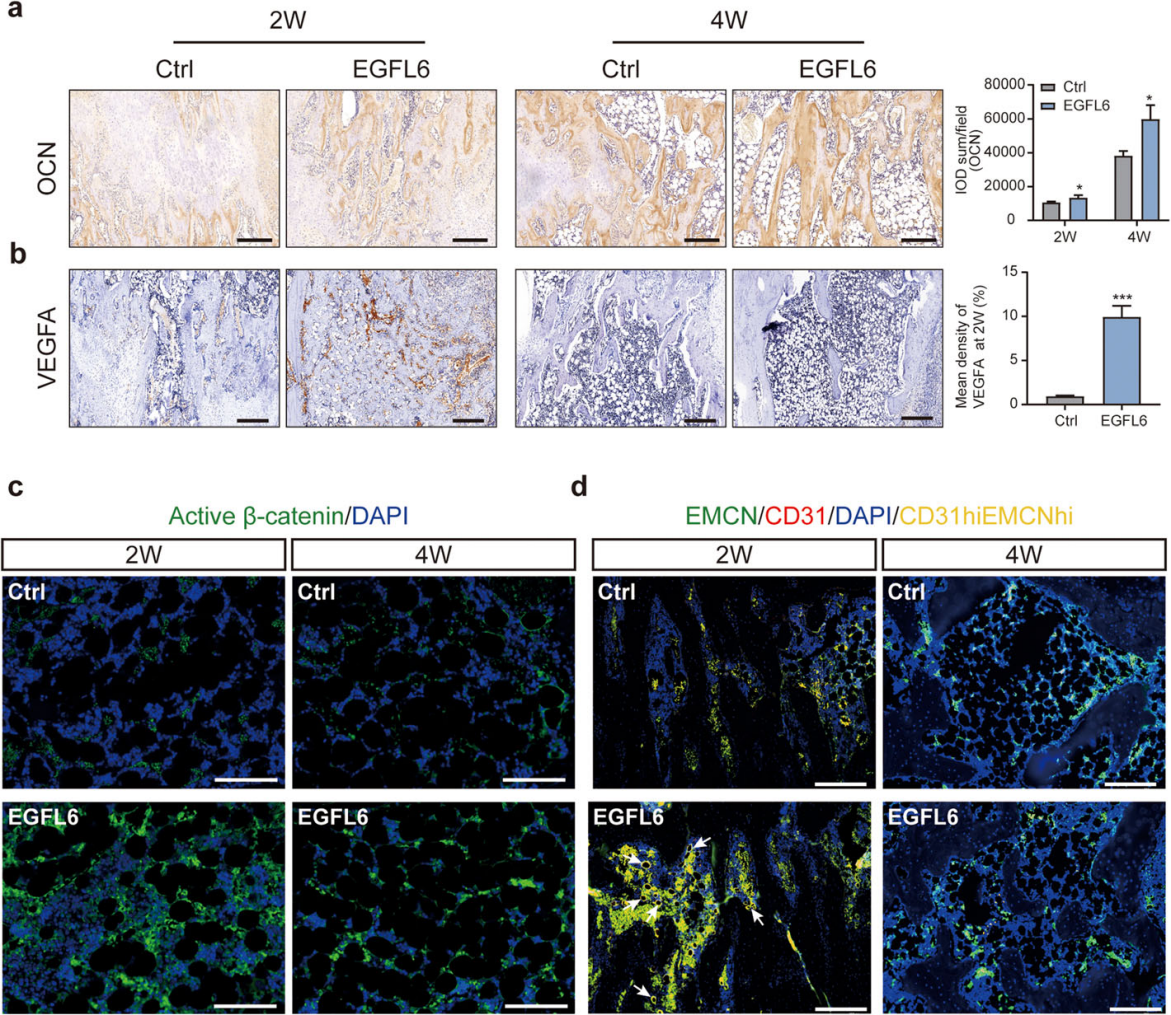
EGFL6 stimulated formation of bone and CD31^hi^EMCN^hi^-positive type H vessels after consolidation for 2 and 4 weeks in a rat tibia DO model. **a** Sections were immunostained for osteocalcin (OCN), a hormone secreted by osteoblasts, and visualized with peroxidase-DAB. Quantitation of OCN-positive staining intensity in the distraction zone is summarized in the histograms on the right. **b** Sections were immunostained for VEGF-A, a key angiogenesis marker, and visualized with peroxidase-DAB. Quantitation of VEGF-A-positive staining intensity in the distraction zone after consolidation for 2 weeks is summarized in the histograms on the right. **c** Immunofluorescent images of regenerated bone sections obtained from the distraction zone immunostained for active β-catenin (green). The sections were counterstained with DAPI (blue), which stains nuclei of all cells. **d** Immunofluorescent images of regenerated bone sections obtained from the distraction zone immunostained for CD31 (red) or endomucin (EMCN, green). The sections were counterstained with DAPI (blue). Note that CD31^hi^EMCN^hi^ (yellow) vessels in EGFL6-treated rats are densely stained (arrows) compared to vessels in the controls. Scale bars for **a–d**, 200 μm. Significant differences were evaluated by Student *t* tests; *p < 0.05

After 2 weeks of consolidation, HE staining revealed that enhanced bone tissue had formed along the columns and in the center of the defect in the EGFL6-treated group. At 4 weeks, the bone marrow cavity had gradually recanalized in the EGFL6 group, occurring rather faster than in the control group (Fig. 6a). Similarly, Masson’s trichrome staining and Safranin O/Fast green staining showed that the interzone contained more mature trabecular bone but fewer fibrous or cartilaginous tissues, respectively, in the EGFL6-treated group compared to that in the control group (Fig. 6b, c). These findings are consistent with those from histological sections immunostained for the osteogenic marker OCN and angiogenic marker VEGF-A (Fig. 7). OCN immunostaining was denser and more widely distributed in sections from the EGFL6-treated group than in those from the control group (Fig. 7a, b). In addition, active β-catenin immunostaining was denser and more widely distributed in the injury zone after EGFL6 treatment (Fig. 7c).

Closer examination of the distraction zone during the consolidation phase revealed that new vessels formed columns alongside the newly developing bone, extending toward the distraction gap. After 2 weeks of consolidation, immunofluorescence staining for CD31 and EMCN showed the presence of CD31^hi^EMCN^hi^-positive type H vessels throughout the distraction gap in the EGFL6-treated group. These findings support the hypothesis that EGFL6 plays a role in developmental and regenerative angiogenesis of type H vessels in bone. However, after 4 weeks of consolidation, in both EGFL6-treated and control rats, the density of CD31^hi^EMCN^hi^-positive endothelium appeared to decline as mature trabecular bone increased (Fig. 7d).

Taken together, in the rat DO model, local infusion of EGFL6 into the distraction zone promoted the formation of CD31^hi^EMCN^hi^-positive endothelial cells over time and widely accelerated bone formation. These results also provide further evidence that angiogenesis and osteogenesis are linked during bone modeling and remodeling [12] and that EGFL6 serves a molecular link between angiogenesis and osteogenesis.

## Discussion

Bone formation is functionally coupled to the vascular network during skeletal system development and postnatal bone repair [3, 9, 46]. Some consider ECs to be important secretory cells in the bone marrow microenvironment, whereas others consider osteoblasts and osteocytes to be crucial in the regulation of bone formation and resorption [37]. However, the cellular and molecular interactions linking angiogenesis and osteogenesis remain obscured. In the present study, we discovered that EGFL6 stimulates bone healing and drives formation of CD31^hi^EMCN^hi^ vasculature, with ECs playing a central role. EGFL6 stimulates both angiogenesis and osteogenic differentiation through Wnt/β-catenin signaling.

Osteoclast lineage cells have been demonstrated to secrete platelet-derived growth factor type BB to recruit osteoprogenitors and ECs to form CD31^hi^EMCN^hi^-positive vessels, linking angiogenesis with osteogenesis [47]. In that study, preosteoclasts were shown to secrete an angiogenic factor that stimulated not only angiogenesis but also supported osteogenesis, functionally coupling the two processes. As osteoclast bone resorption and osteoblast bone formation function cooperatively during bone remodeling [48], we reasoned that, in the same way, other factors could interact with BMSC/osteoblast cell lines and ECs. One of these factors appears to be EGFL6, an important piece of the puzzle that connects angiogenesis-osteogenesis coupling in bone regeneration.

In our study, EGFL6 stimulated both ECs and BMSCs in vitro, with EGFL6 promoting EC proliferation, migration, and increased vascularization. These results are consistent with previous findings [41, 49]. RT-PCR and western blot analyses showed that EGFL6 upregulates both CD31 and EMCN expression in ECs, providing evidence for its potential role in promoting the formation of CD31^hi^EMCN^hi^-positive endothelium. As EGFL6 is expressed in osteoblastic-like cells, not ECs [28], EGFL6 may regulate angiogenesis via a paracrine mechanism, acting between osteoblasts and ECs in the bone microenvironment [38].

In cases of bone defect repair, BMSCs could generate osteoblasts and their progenitors, which contribute to bone homeostasis and fracture healing. As BMSCs are widely used in basic research and for clinical applications [50, 51], molecules that can create an optimal osteogenic microenvironment and enhance BMSC function are of great value [52]. We discovered that EGFL6 strongly induced BMSC osteogenesis in vitro in a time- and concentration-dependent manner. The osteogenic differentiation capacity of EGFL6-treated BMSCs was much greater than that of untreated BMSCs (control), as demonstrated by expression of BMP2, RUNX2, and CXCR4. BMSCs genetically engineered to overexpress BMP2 or CXCR4 increase not only bone strength but also promote bone regeneration [53, 54]. These findings suggested the possibility of a positive feedback loop, wherein osteoblast-like cells secrete EGFL6 and EGFL6 promotes BMSCs differentiation. A mesenchymal stem cell (MSC) population resides in the perivascular niche of the bone marrow [55, 56]. Other than their capacity to transdifferentiate or differentiate into osteoblasts, BMSCs themselves can communicate with ECs and integrate into the bone regeneration framework [57, 58]. At optimal EGFL6 concentrations, VEGF-A expression in BMSCs was significantly enhanced compared to untreated BMSCs. These results indicate that via EGFL6, the interaction of BMSCs with ECs further regulated angiogenesis through the secretion of angiogenic growth factors [2].

In the tight coupling of angiogenesis and osteogenesis, accumulating evidence indicates that several signaling pathways are potentially activated, including Notch signaling, Hif1a/VEGF signaling, and TGFβ/Smad signaling [9, 59, 60]. In the present study, we demonstrated for the first time that EGFL6 stimulated both angiogenesis and osteogenesis through the Wnt/β-catenin pathway. This pathway is an essential signaling axis in stem cell proliferation, differentiation, and tissue homeostasis during development [61, 62]. A range of Wnt ligands mediates cell-to-cell communication and adhesion, while β-catenin functions as the main downstream effector in this axis [63]. The regulation of the Wnt/β-catenin pathway is important for osteoblast differentiation and bone regeneration [39]. Once osteogenic differentiation is initiated, high β-catenin levels are needed to promote osteogenesis but prevent chondrogenesis.

In the present study, we observed higher expression of β-catenin and downstream osteogenic markers in EGFL6-treated cells maintained in vitro. DKK1, an inhibitor of Wnt/β-catenin signaling, only partially blocked the EGFL6-mediated increase we observed in osteogenesis, indicating that additional pathways are likely involved in the EGFL6-mediated activity. At the same time, Wnt/β-catenin pathway signaling was also critically involved in the modulation of EC migration and vascular sprouting. Wnt/β-catenin signaling is also fundamental to normal CNS vascularization [64], as well as vascularization in chondrogenesis [65]. Here, we observed increased expression of β-catenin in HUVECs following EGFL6 treatment in vitro, suggesting that Wnt and downstream β-catenin signaling are potential functional targets involved in angiogenesis-osteogenesis coupling.

We further investigated the effects of EGFL6 in vivo in a rat tibia DO model*.* Following osteotomy, new bone is formed within the distraction gap, bridging the two bone segments [44, 45]. Compared to other models, DO mirrors temporal and spatial bone remodeling pathology with a much greater angiogenic response, making it an attractive model for investigating the effects of EGFL6 on bone regeneration that is accompanied by improved vascularization, extensive mineralization, and eventual trabecular remodeling.

**Fig. 8:**
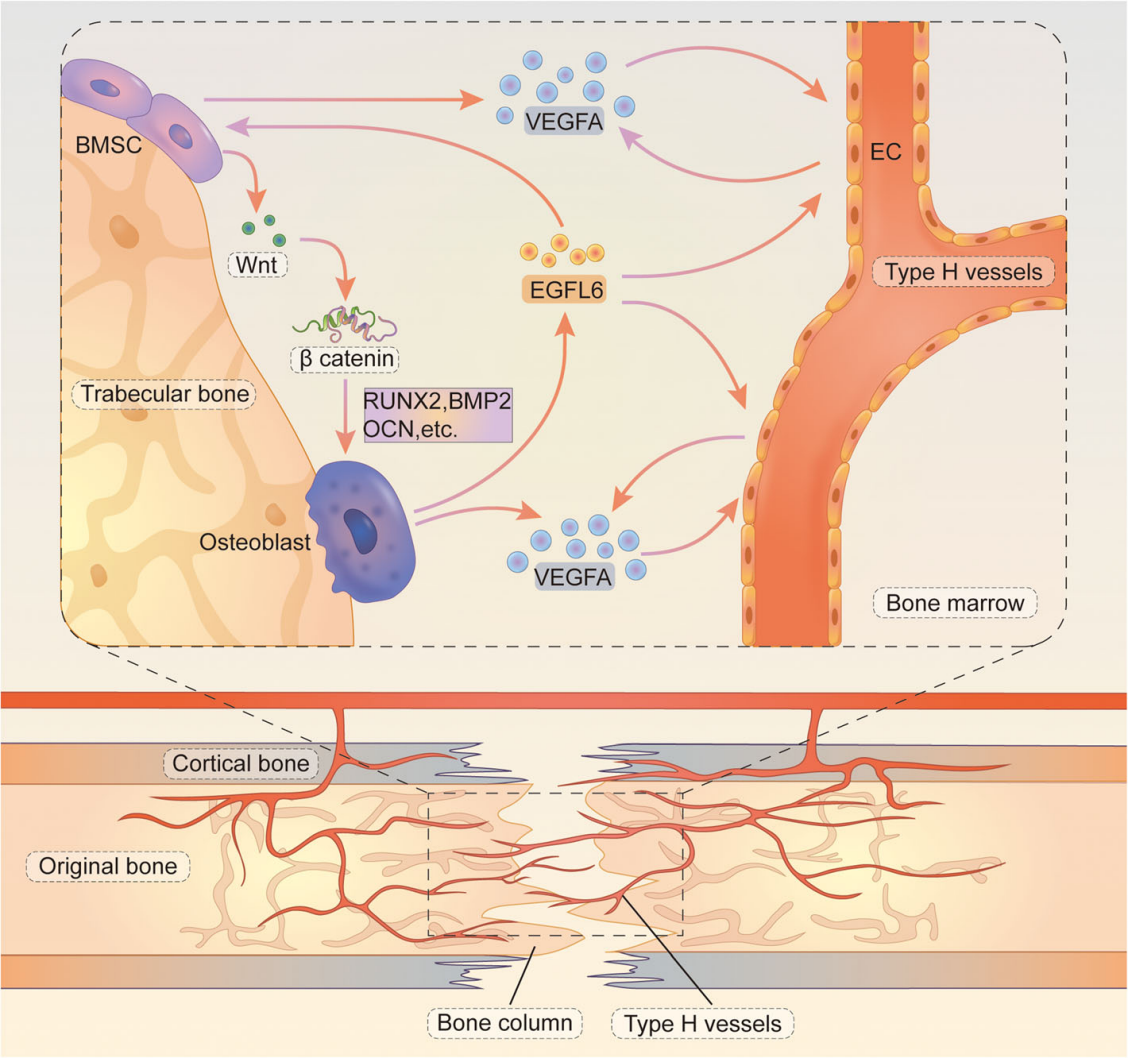
Working model of EGFL6-mediated signaling, illustrating the coupling of angiogenesis and osteogenesis in the rat DO model. During the consolidation phase of bone remodeling, type H vessels form alongside newly developing bone and extend toward the distraction gap. In the bone marrow microenvironment, multiple cell types secrete angiogenic factors to support type H vessel formation. Osteoblast-lineage cells and ECs secrete VEGF-A. EGFL6 secreted by osteoblasts enhances VEGF-A expression in ECs to promote cell migration, tube formation, and branching, which further stimulates the formation of type H vessels during early consolidation phase. As a key regulatory factor, EGFL6 also promotes osteogenic differentiation of BMSCs into osteoblast-lineage cells, activated by the Wnt/β-catenin signaling pathway. EGFL6 also increases expression of the osteogenic proteins RUNX2, BMP2, and OCN, leading to faster restoration of the bone defect in the DO model. Abbreviations: ECs, endothelial cells; BMSCs, bone marrow mesenchymal stem cells; EGFL6, epidermal growth factor-like domain-containing protein 6; VEGF-A, vascular endothelial growth factor; RUNX2, Runt-related transcription factor 2; BMP2, bone morphogenetic protein 2; OCN, osteocalcin

Figure 8 presents our working model of EGFL6-mediated signaling in bone repair. The schematic illustrates the coupling of angiogenesis and osteogenesis in a rat DO model. The direct infusion of EGFL6 into the distraction gap accelerated bone mineralization and recanalization during the consolidation phase. Micro-CT analysis indicated that newly formed bone in EGFL6-treated rats was more mineralized than that in untreated control rats. Simultaneously, EGFL6 enhanced the formation of type H vessels along the primary mineralization matrix, particularly during the early part of the consolidation phase. The increase in CD31^hi^EMCN^hi^-positive ECs indicated that EGFL6 may regulate the coupling of angiogenesis with bone formation and that EGFL6 plays a key role in trabecular bone remodeling. This finding has important implications for certain conditions like compromised fracture healing and for treatments to repair bone defects.

Our results are subject to some limitations. As we observed a highly selective distribution of CD31^hi^EMCN^hi^-positive vessels at the distal end of the arterial network in vivo in EGFL6-treated rats of the DO experiment, it is likely that these vessels influenced bone regeneration and metabolism of other cell types that we did not study. Thus, we cannot exclude the possibility that these other unidentified cell types contributed to EGFL6-associated enhancement of osteogenesis. Although we demonstrated the involvement of EGFL6 in the coupling between angiogenesis and osteogenesis, the detailed mechanisms remain to be elucidated. Also, future studies should consider using other animal models besides the tibia DO model with EGFL6 infusion, such as EGFL6-knockout mice or alternative application methods, to test the therapeutic effects of EGFL6.

Another possible issue for future study is why ECs migrated more effectively at 200 and 500 ng/ml EGFL6 but capillary tube formation was more significant at 50 ng/ml EGFL6 and to a lesser extent at 200 ng/ml EGFL6 and not near as effectively with 500 ng/ml. The optimal concentration of EGFL6 may be different depending on the cell state and the microenvironment. We also observed differences in mRNA expression and what one might expect in the corresponding protein expression. These could be accounted for by transcriptional or post-transcriptional regulation.

## Conclusions

The present study suggests that EGFL6 is a key player in the tight, functional coupling of angiogenesis and osteogenesis, possibly via the Wnt/β-catenin pathway activation and stimulation of CD31^hi^EMCN^hi^ type H vessels. Boosting concentrations of EGFL6 and/or other vascular-targeted factors may be a new strategy for the treatment of compromised fracture healing and bone defect restoration. Moreover, this enhanced understanding of the role of EGFL6 angiogenesis-osteogenesis coupling in the bone microenvironment may help to develop new diagnostic biomarkers and therapies for bone pathologies like osteoporosis and osteonecrosis.

## Supplementary Information

**Additional file 1. **legend Involvement of other potential signaling pathways in EGFL6-mediated angiogenesis and osteogenesis is supported by protein analysis of treated EC cells maintained *in vitro*. (**a**) Western blots of cell lysates from EGFL6-treated HUVECs probed with antibodies against Akt, P-Akt, and P-ERK1/2. The cultured cells were treated with 0, 50, 200, or 500 ng/ml EGFL6, and then harvested for protein content. (**b, c**) Western blots of lysates from EGFL6-treated BMSCs probed with antibodies against Akt and P-Akt. BMSCs underwent osteogenic induction with 0, 50, 200, or 500 ng/ml EGFL6 for 5 days (**b**) or 10 days (**c**). GADPH is the loading control.

**Additional file 2. **legend Rat tibial distraction osteogenesis (DO) model. Representative photographs (ventral views) illustrating steps of the surgical procedure to implant the distraction device (see the Methods for details). The monolateral external fixator device is shown *in situ* in panels g and h. Animal care and procedures were approved by the Animal Care and Use Committee of Shanghai Jiao Tong University Affiliated Sixth People's Hospital.

## Data Availability

The datasets used and/or analyzed during the current study are available from the corresponding author upon reasonable request.

## Abbreviations

EGFL6: Epidermal growth factor-like domain-containing protein 6
HUVEC: Human umbilical cord vein endothelial cell
ALP: Alkaline phosphatase
AR-S: Alizarin Red S
BMSC: Bone marrow mesenchymal stem cell
DO: Distraction osteogenesis
EC: Endothelial cell
VEGF-A: Vascular endothelial growth factor-A
EGF: Epidermal growth factor
CCK-8: Cell Counting Kit-8
PFA: Paraformaldehyde
ECTFA: EC tube-formation assay
DKK1: Dickkopf-related protein 1
BV/TV: Bone volume/total volume
BMD: Bone mineral density
OCN: Osteocalcin
HE: Hematoxylin and eosin
PBS: Phosphate-buffered saline
qRT-PCR: Quantitative reserve-transcriptase polymerase chain reaction
SD: Standard deviation
OIM: Osteogenic induction medium
