# Supplementary material for: EGFL6 regulates angiogenesis and osteogenesis in distraction osteogenesis via Wnt/β-catenin signaling
Source: Stem Cell Res Ther. 2021 Jul 22;12:415. doi: 10.1186/s13287-021-02487-3 (PMC8296592; doi:10.1186/s13287-021-02487-3)
Supplement: Supplementary file 1 — Additional file 1. legend Involvement of other potential signaling pathways in EGFL6-mediated angiogenesis and osteogenesis is supported by protein analysis of treated EC cells maintained in vitro. (a) Western blots of cell lysates from EGFL6-treated HUVECs probed with antibodies against Akt, P-Akt, and P-ERK1/2. The cultured cells were treated with 0, 50, 200, or 500 ng/ml EGFL6, and then harvested for protein content. (b, c) Western blots of lysates from EGFL6-treated BMSCs probed with antibodies against Akt and P-Akt. BMSCs underwent osteogenic induction with 0, 50, 200, or 500 ng/ml EGFL6 for 5 days (b) or 10 days (c). GADPH is the loading control. [file 13287_2021_2487_MOESM1_ESM.docx]

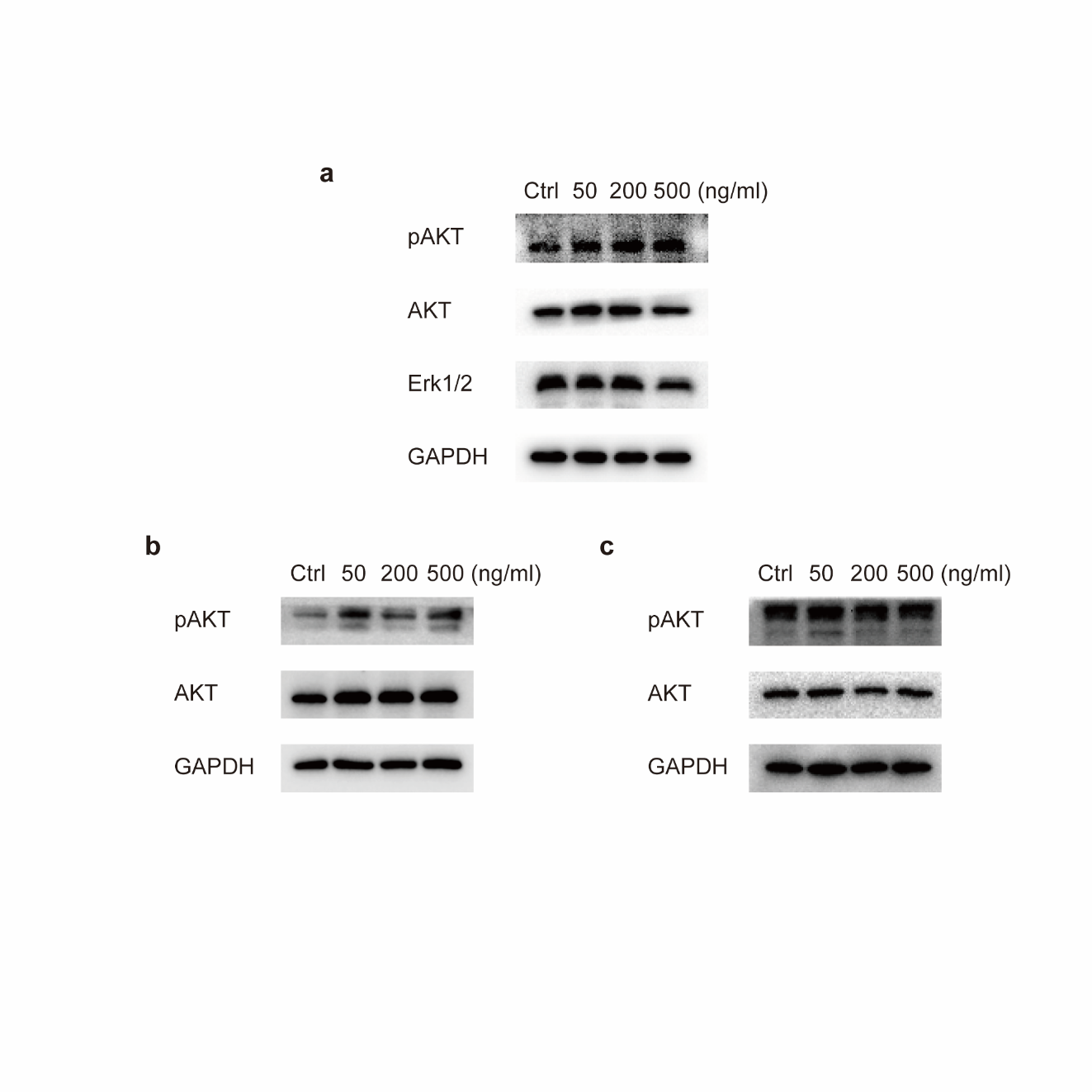


**Additional file 1** **legend** Involvement of other potential signaling pathways in EGFL6-mediated angiogenesis and osteogenesis is supported by protein analysis of treated EC cells maintained *in vitro*. (**a**) Western blots of cell lysates from EGFL6-treated HUVECs probed with antibodies against Akt, P-Akt, and P-ERK1/2. The cultured cells were treated with 0, 50, 200, or 500 ng/ml EGFL6, and then harvested for protein content. (**b, c**) Western blots of lysates from EGFL6-treated BMSCs probed with antibodies against Akt and P-Akt. BMSCs underwent osteogenic induction with 0, 50, 200, or 500 ng/ml EGFL6 for 5 days (**b**) or 10 days (**c**). GADPH is the loading control.
