# Supplementary material for: EGFL6 regulates angiogenesis and osteogenesis in distraction osteogenesis via Wnt/β-catenin signaling
Source: Stem Cell Res Ther. 2021 Jul 22;12:415. doi: 10.1186/s13287-021-02487-3 (PMC8296592; doi:10.1186/s13287-021-02487-3)
Supplement: Supplementary file 2 — Additional file 2. legend Rat tibial distraction osteogenesis (DO) model. Representative photographs (ventral views) illustrating steps of the surgical procedure to implant the distraction device (see the Methods for details). The monolateral external fixator device is shown in situ in panels g and h. Animal care and procedures were approved by the Animal Care and Use Committee of Shanghai Jiao Tong University Affiliated Sixth People's Hospital. [file 13287_2021_2487_MOESM2_ESM.docx]

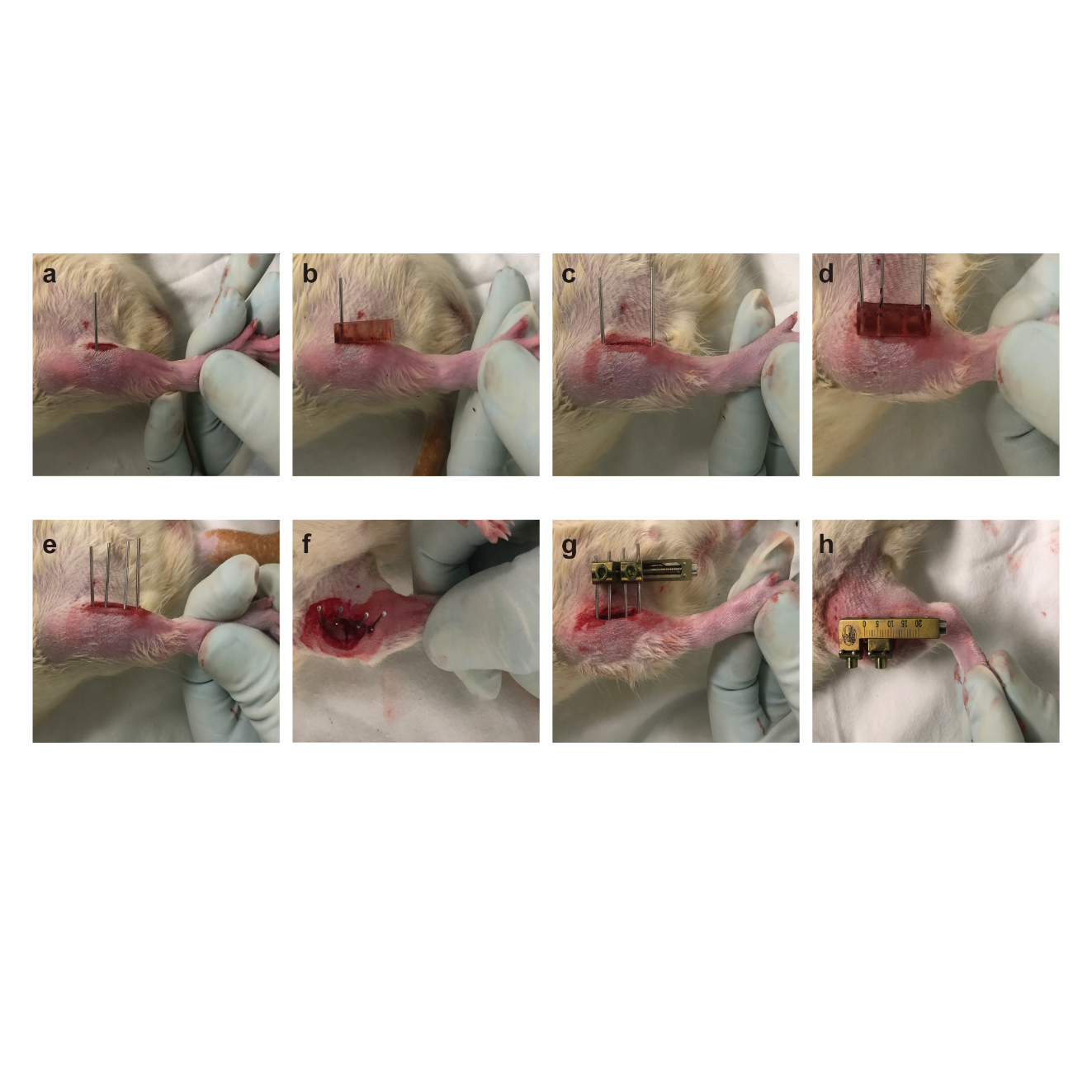


**Additional file 2** **legend** Rat tibial distraction osteogenesis (DO) model. Representative photographs (ventral views) illustrating steps of the surgical procedure to implant the distraction device (see the Methods for details). The monolateral external fixator device is shown *in situ* in panels g and h. Animal care and procedures were approved by the Animal Care and Use Committee of Shanghai Jiao Tong University Affiliated Sixth People's Hospital.
